# Consumo infantil de alimentos: ¿relación con el estado nutricional materno?

**DOI:** 10.15649/cuidarte.2038

**Published:** 2023-03-29

**Authors:** Ana Clara da Cruz-Della-Torre, Thais da Silva-Maciel, Débora Vasconcelos-Bastos-Marques, Tábatta Renata-Pereira-de-Brito, Daniela Braga-Lima

**Affiliations:** 1 Universidade Federal de Alfenas, Alfenas, Minas Gerais, Brasil. E-mail: ana.torre@sou.unifal-mg.edu.br Autor de correspondencia Universidade Federal de Alfenas Universidade Federal de Alfenas Brazil ana.torre@sou.unifal-mg.edu.br; 2 Universidade Federal de Alfenas, Alfenas, Minas Gerais, Brasil. E-mail: thais.maciel@sou.unifal-mg.edu.br Universidade Federal de Alfenas Universidade Federal de Alfenas Brazil thais.maciel@sou.unifal-mg.edu.br; 3 Universidade Federal de Alfenas, Alfenas, Minas Gerais, Brasil. E-mail: deboravbastos@gmail.com Universidade Federal de Alfenas Universidade Federal de Alfenas Brazil deboravbastos@gmail.com; 4 Universidade Federal de Alfenas, Alfenas, Minas Gerais, Brasil. E-mail: tabatta_renata@hotmail.com Universidade Federal de Alfenas Universidade Federal de Alfenas Brazil tabatta_renata@hotmail.com; 5 Universidade Federal de Alfenas, Alfenas, Minas Gerais, Brasil. E-mail: danibraga@unifal-mg.edu.br Universidade Federal de Alfenas Universidade Federal de Alfenas Brazil danibraga@unifal-mg.edu.br

**Keywords:** Mother-Child Relations, Maternal Nutrition, Child Health, Nutritional Status, Food Consumption., Relagóes Máe-Filho, Nutrigáo Materna, Saúde da Crianga, Estado Nutricional, Consumo de Alimentos., Relaciones madre-hijo, Nutrición Materna, Salud infantil, Estado nutricional, Consumo de alimentos.

## Abstract

**Introducción:**

La figura materna tiene una fuerte influencia en la salud del niño, el estado nutricional y la formación de los hábitos alimentarios del niño, ya que es la principal cuidadora de su hijo.

**Objetivo:**

determinar el estado nutricional de las madres y su asociación con el consumo de alimentos de los niños.

**Materiales y Métodos:**

Estudio transversal realizado con 163 binomios madre-hijo menores de 24 meses atendidos en Unidades de Salud de la Familia. Se utilizó un cuestionario para recolectar las variables sociodemográficas y antropométricas de madres e infantes. El estado nutricional de los lactantes se clasificó por el índice de masa corporal por indicador de edad y el diagnóstico del estado nutricional de las madres por el índice de masa corporal. La práctica de alimentación del lactante se analizó utilizando formas de marcadores de consumo de alimentos propuesto por el Ministerio de Salud de Brasil.

**Resultados:**

Se observó que el 51,53% de las madres tenían sobrepeso y el 30,06% de los niños tenían sobrepeso, según IMC/Edad. En cuanto al consumo de alimentos infantiles, hubo una marcada presencia de alimentos ultraprocesados. El estado nutricional materno inadecuado se asoció con el consumo de snacks envasados el día anterior a la encuesta (p = 0,002).

**Conclusión:**

El perfil materno tiene una gran influencia en el consumo de alimentos del lactante, por lo que es necesario implementar actividades de educación en salud para asesorar a las familias, reforzando la importancia de introducir adecuadamente los alimentos complementarios.

## Abstract (en)

**Introduction:**

As the primary caregiver of her child, the mother figure has a strong influence on the child's health, nutritional status, and formation of eating habits.

**Objective:**

To determine the nutritional status of mothers and its association with children's food consumption.

**Objective:**

To determine the nutritional status of mothers and its association with children's food consumption. A cross-sectional study was conducted with 163 mother-child pairs at <24 months of age attended in Family Health Units.

**Materials and Methods:**

A questionnaire was used to collect sociodemographic and anthropometric variables of mothers and infants. The nutritional status of the infants was determined using the body mass index by age; the diagnosis of the nutritional status of the mothers was determined by body mass index. Infant feeding practice was evaluated using the Food Intake Markers proposed by the Brazilian Ministry of Health.

**Results:**

51.53% of the mothers were overweight, and 30.06% of the children were overweight, according to BMI/age. In terms of children's food consumption, there was a marked presence of ultra-processed foods. Inadequate maternal nutritional status was associated with consuming packaged snacks the day before the survey (*p* = 0.002).

**Conclusion:**

The maternal profile significantly influences infant food consumption; therefore, it is necessary to implement health education activities to advise families and reinforce the importance of adequately introducing complementary foods.

## Resumo

**Introdujo:**

A figura materna exerce forte influencia na saúde da crianza, no estado nutricional e na formagáo dos hábitos alimentares da crianza, visto que ela é a principal cuidadora de seu filho.

**Objetivo:**

determinar o estado nutricional de máes e sua associagáo com o consumo alimentar de crianzas.

**Materiais e Métodos:**

Estudo transversal realizado com 163 duplas máe- filho menores de 24 meses atendidas em Unidades de Saúde da Família. Um questionário foi utilizado para coletar as variáveis sociodemográficas e antropométricas de máes e bebes. O estado nutricional dos lactentes foi classificado pelo índice de massa corporal por indicador de idade e o estado nutricional das máes diagnosticadas pelo índice de massa corporal. A prática de alimentado infantil foi analisada por meio de formulários de marcadores de consumo alimentar propostos pelo Ministério da Saúde do Brasil.

**Resultados:**

Observou-se que 51,53% das máes estavam acima do peso e 30,06% das crianzas estavam acima do peso, segundo IMC/ Idade. Em relagáo ao consumo de alimentos infantis, houve presenta marcante de alimentos ultraprocessados. O estado nutricional materno inadequado foi associado ao consumo de salgadinhos embalados no dia anterior a pesquisa (p = 0,002).

**Conclusao:**

O perfil materno tem grande influencia no consumo alimentar do lactente, por isso é necessário implementar agóes de educado em saúde para orientar as familias, reforjando a importancia da introdugáo adequada dos alimentos complementares.

## Introducción

La prevalencia del sobrepeso/obesidad en los niños ha sido un problema importante de salud pública en las últimas décadas en todo el mundo, incluyendo en Brasil^1^^,^^2^. Datos del Estudio de Alimentación y Nutrición Infantil (ENANI-2019), apuntaron una prevalencia de sobrepeso de 10,1% en menores de 5 años, mientras que el sobrepeso materno se encontró en más de la mitad de la población estudiada (58,6%^3)^. Por lo tanto, ante esta situación es necesario una acción conjunta de los diferentes niveles de gobierno, tanto regionales como locales, fomentando iniciativas de afrontamiento en la prevención y control del sobrepeso/obesidad^1^. Además, los niños con sobrepeso de hoy en día son más propensos a convertirse en adultos obesos^4^.

Tradicionalmente, el análisis del consumo de alimentos se basa en los nutrientes y el contenido energético presentes en los alimentos, sin embargo, la Organización Mundial de la Salud (OMS) determina que el consumo de alimentos puede evaluarse de acuerdo con los alimentos ingeridos, y no solo por los nutrientes presentes en ellos. por lo tanto, los estudios que utilizan cuestionarios que mencionan el consumo habitual de alimentos pueden utilizarse para identificar patrones dietéticos^5^. Así, con el fin de mejorar las condiciones de nutrición, salud y alimentación de la población, el Sistema de Vigilancia Alimentaria y Nutricional (SISVAN) a través de sus formularios, principalmente marcadores de consumo de alimentos, permiten dicho análisis de los alimentos^6^.

La salud de un niño depende en gran medida de los hábitos nutricionales que se forman en las primeras etapas de su vida^7^ Así, la familia ejerce un importante papel en la formación de hábitos alimenticios infantiles, ya que es el principal agente en el proceso de socialización, transformación e implementación de estilos de vida para los infantes^8^. Dentro de la familia, la madre tiene el papel fundamental en la construcción de hábitos alimenticios de los niños, ya que además de ser la cuidadora principal del niño, ella decide cómo se alimentará al niño^9^^-^^11^.

Además, los estudios indican que la forma de cuidado de la madre está directamente relacionada con su nivel educativo, las orientaciones recibidas por profesionales de la salud y/o los medios de comunicación, el estrés psicológico, el estado nutricional, el apoyo social y el tiempo disponible para dedicarse al cuidado infantil^12^^-^^14^.

Entre los factores de riesgo modificables asociados con la obesidad infantil se encuentran la ingesta de alimentos y el estilo de vida, y las intervenciones preventivas deben estar dirigidas a cambiar estos factores, tales como: los padres pueden controlar las elecciones de alimentos de sus hijos, restringiendo el consumo de alimentos con alto contenido de azúcar, evitando la presión de comer más , o usar la comida como recompensa por el buen comportamiento, controlar el tiempo frente a la pantalla^15^.

Dada la importancia de la figura materna en el estado nutricional de sus hijos y su posible impacto en la alimentación ofrecida al niño y en la identificación y manejo del sobrepeso y la obesidad infantil, el objetivo de este estudio fue determinar el estado nutricional de las madres y su asociación con el consumo de alimentos de los niños.

## Materiales y Métodos

### Participantes

Este estudio de corte transversal se llevó a cabo con una muestra compuesta por un binomio madre-hijo, representado por niños de entre 6 y 24 meses de vida y sus respectivas madres, seguido en unidades de salud familiar de un pequeño municipio ubicado en la región sur de Minas Gerais en 2019, en los meses de marzo a diciembre.

La muestra se calculó mediante el programa OpenEpi®, utilizando la ecuación: n= [EDFF*Np(1-p)]/ [(d2 /Z2 1-a/2*(N -1)+p*(1- p )], donde: N= tamaño de la población (para el factor de corrección de población finita o fcp); p= % de frecuencia hipotética del factor de resultado en la población; EDFF= efecto de diseño para encuestas grupales y d= límites de confianza como % de 100 (absoluto +/-%).Para el cálculo se consideró el promedio de nacidos vivos residentes en Alfenas - MG en los años 2017 (991) y 2018 (998^16)^, intervalo de confianza del 95 %, error de muestreo del 5 % y una estimación del 50% para la prevalencia del evento estudiado (destete precoz, prácticas inadecuadas de alimentación^17^^,^^18^. Además, se estableció un aumento del 20% para compensar las posibles pérdidas y 344 niños participaron en el estudio^19^. Con base en estos datos, la muestra estuvo integrado por 163 niños y sus respectivas madres, la madre de cada participante firmó por escrito el consentimiento informado antes de finalizar la inversión.

Los criterios de exclusión fueron niños que no vivían en el mismo hogar que sus madres, ya que ellas eran las responsables de responder el cuestionario. Además, se excluyeron los niños que presentaban alguna patología o inmovilizaciones que impidieran la medición del peso y la talla y los menores de 6 meses y mayores de 24 meses.

### Variables

La recopilación de datos se produjo a través de la aplicación de un cuestionario que presentaba variables sociodemográficas y antropométricas de la madre, y el estado nutricional del niño. El estado nutricional del niño se caracterizó por el perfil antropométrico y los marcadores de la ingesta de alimentos. El análisis del consumo de alimentos se realizó mediante un cuestionario sobre marcadores de consumo de alimentos de niños menores de dos años, para los efectos del Sistema de Vigilancia Alimentaria y Nutricional del Ministerio de Salud de Brasil, consideradas como variables independientes^20^^,^^21^.

Para evaluar el estado nutricional de los niños, se utilizaron medidas como el peso y la longitud de acuerdo con la metodología propuesta por el Ministerio de Salud^21^. Para evaluar el estado nutricional de los bebés, se utilizó el indicador del índice de masa corporal por edad (IMC/edad). Los datos antropométricos recogidos fueron analizados del programa Anthro 3 de la OMS^22^, y así estableció el diagnóstico nutricional, expresado en z score, y en comparación con los patrones de crecimiento propuestos por la Organización Mundial de la Salud (OMS^22)^. En este estudio, para el indicador de IMC/edad, las categorías “bajo peso”, “riesgo de sobrepeso” y “obesidad” se agruparon en la misma categoría llamada “inadecuada”; y la clasificación “eutrofia” se clasificó como “adecuada”.

Para medir el estado nutricional materno, las mediciones antropométricas recogidas incluyeron el peso y la altura por la metodología también adoptada por el Ministerio de Salud y evaluada por el Índice de Masa Corporal (IMC), utilizando los puntos de corte propuestos por el Ministerio de Salud^21^ Posteriormente, estos puntos de corte se dividieron en dos categorías: “adecuada”, que comprendía eutrofia, e inadecuada, representada por la desnutrición, el sobrepeso, la obesidad grado I, II y III. A efectos de análisis, esta variable se utilizó como dependiente. 

### Análisis Estadístico

Los datos se introdujeron en el programa de Excel y más tarde se analizaron en el programa *paquete estadístico para las ciencias sociales* (SPSS), versión 20.0. La normalidad de la distribución de datos se analizó utilizando la *prueba Shapiro-Wilk* en un 5%. En el análisis descriptivo de los datos, se estimaron distribuciones de frecuencia, medios y desviaciones estándar para las variables continuas del estudio; para variables categóricas, se estimaron distribuciones de frecuencia. La asociación entre las variables fue probada por el cruce de variables utilizando la prueba Z|Chi-Square o la exacta del *pescador,* con sus respectivos intervalos de confianza del 95% (IC del 95%).

### Aspectos Éticos

El presente estudio se realizó con parte de los datos del proyecto de investigación más amplio titulado “Consumo de alimentos en la primera infancia: contribución a los estudios de vigilancia alimentaria y nutricional”, aprobado por el Comité de Ética de investigación de la Universidad Federal de Alfenas- UNIFAL/MG (CAAE: 06262819.4.0000.5142/ protocolo n° 3.199.539/2019). 

## Resultados

El estudio incluyó a 163 bebés de entre 6 y 24 meses. La edad media de los niños era de 13,04 (± 4,70) meses y la edad materna media era de 30,14 (± 6,88 años). En cuanto al estado nutricional, el 30,06% de los niños tenían sobrepeso e y el 10,43% tenían bajo peso, según el IMC/Edad. Mientras que la prevalencia en la madre de un estado nutricional inadecuado fue del 56,44%, siendo sólo el 4,91% bajo peso y el 51,53% sobrepeso (sobrepeso y obesidad entre las madres fue del 28,83% y del 22,70%, respectivamente). De las madres que participaron en el estudio, más de la mitad tenían ingresos familiares inferiores a dos salarios mínimos (59,03%) y el 61,73% informó tener menos de ocho años de estudio (Tabla 1).

**Tabla 1:** Características demográficas, socioeconómicas y antropométricas de los niños de entre seis y 24 meses y sus madres, Alfenas - MG, 2019.

variable	N° 163	%	IC 95%
Edad del niño (meses) - media± SD	13,04±4,70		
Sexo - n (%)			
femenino	75	46,01	38,19 - 53,99
masculino	88	53,99	46,01 - 61,81
Estado nutricional del niño - n (%)			
Bajo peso	17	10,43	6,19 - 16,17
Eutro fi a	97	59,51	51,55 - 67,12
sobrepeso	49	30,06	23,14 - 37,73
Edad de la Madre (años) - media± SD	30,14± 6,88		
La educación de la madre - n (%)^a^			
< 8 años	100	61,73	53,78 - 69,24
> 8 años	62	38,27	30,76- 46,22
Ingresos - n (%)^b^			
*< 2* salarios mínimos	85	59,03	50,53 - 67,15
> 2 salarios mínimos	59	40,97	32,85 - 49,47
Diagnóstico Nutricional Materno - n (%)			
Bajo peso	8	4,91	2,14 - 9,44
Eutrofia	71	43,56	35,82 - 51,54
Sobrepeso	84	51,53	43,59 - 59,42

IC (95%): Intervalo de confianza de 95% da %. ^a^ n=162; ^b^n=144 Fuente: Elaboración de los autores

Al investigar la asociación (P<0.05) entre las variables de ingesta demográficas, antropométricas y alimentarias de niños con un estado nutricional materno inadecuado, no se encontraron relaciones. Sin embargo, los hallazgos muestran la presencia de todos los grupos alimentarios: cereales, legumbres, verduras y verduras, frutas y carnes y huevos en la alimentación de los lactantes independientemente del estado nutricional materno. También cabe destacar que menos de la mitad de los bebés (47,24%) tuvieron lactancia materna exclusiva más de 150 días comparados con aquellos que tenían la AME ofrecida con una duración inferior a 150 días (Tabla 2).

**Tabla 2:** Caracterización y análisis de la ingesta saludable de alimentos desde el día anterior a la encuesta de niños mayores de seis meses según el estado nutricional materno, Alfenas - MG, 2019.

Variable	Ingesta de alimentos saludables			Clasificación del estado nutricional materno				*Valor-p*
	total			Adecuado		inadecuado		
	N	% IC (95%)		N %		N %		
Estado nutricional infantil								0,935
inadecuado	66	40,49	32,88 - 48,45	29	43,94	37	56,06	
Adecuado	97	59,51	51,55 - 67,12	42	43,30	55	56,70	
Sexo								0,673
Masculino	88	53,99	46,01 - 61,81	37	42,05	51	57,95	
Hembra	75	46,01	38,19 - 53,99	37	49,33	41	54,67	
¿El niño recibió ayer leche								0,194
materna?								
No	92	56,44	48,46 - 64,18	36	39,13	56	60,87	
Sí	71	43,56	35,82 - 51,54	35	49,30	36	50,70	
Lactancia materna exclusiva por edad (AME)											0,786
< 30 días	43	26,38	19,80 - 33,85	17	39,53	26	60,47	
> 31 días < 150 días	43	26,38	19,80 - 33,85	21	48,84	22	51,16	
> 150 días	77	47,24	39,38 - 55,20	33	42,86	44	57,14	
El niño recibió ayer:								
Preparaciones lácteas								0,500
> 2 botellas	128	78,53	71,42 - 84,57	54	42,19	74	57,81	
< 2 botellas	35	21,47	15,43 - 28,58	17	48,57	18	51,43	
Fruta entera*	149	91,98	86,67 - 95,66	66	44,30	83	55,70	0,345
Comida de Sal*	158	97,53	93,80 - 99,32	68	43,03	90	56,96	0,781
Legumbres*	144	88,34	82,40 - 92,83	62	43,06	82	56,94	0,722
Carne y/o huevo*	137	84,05	77,51 - 89,31	58	42,34	79	57,66	0,470
Frijoles*	148	90,80	85,28 - 94,46	62	41,89	86	58,11	0,178
Cereales*	150	92,60	87,42 - 96,11	65	43,33	85	56,67	0,911

C I (95%): Intervalo de confianza del 95 % del %; * variable “no”, presentaron datos de la variable “sí” que representa el consumo de alimentos.

*Fuente: Elaboración de los autores*

En cuanto a la ingesta insalubre de alimentos de los bebés, fue posible observar la ingesta de alimentos ultraprocesados en el día anterior y en el último mes de la investigación, así como la introducción temprana de otros alimentos. Encontró una asociación entre la ingesta de aperitivos y el estado nutricional materno inadecuado (p=0,002) (Tabla 3).

Sin embargo, cabe destacar que la prevalencia del consumo de azúcar, comer mientras veía la televisión, el consumo de gachas o leche espesada con harinas, jugo industrializado y refrescos eran mayores entre los niños que las madres que tenían un estado nutricional inadecuado.

Además, se observó una introducción temprana de miel, azúcares y alimentos antes de los seis meses en estos niños estudiados (Tabla 3).

**Tabla 3:** Caracterización y análisis de la ingesta de alimentos no saludables y hábito de comer viendo televisión el día anterior a la encuesta en niños mayores de seis meses según estado nutricional materno, Alfenas - MG, 2019

Variable*		Ingesta de alimentos no saludables			Clasificación del estado nutricional materno			*Valor-*
		total			Adecuado	inadecuado		
	N	%	IC (95%)	N	%	N	%	
El niño recibió ayer:								
Embutidos	17	10,43	6,19 - 16,17	8	47,06	9	52,94	0,758
Galletas, rellenas /dulces/golosinas	49	30,25	23,29 - 37,95	22	44,90	27	55,10	0,856
Fideos instantáneos/Snack/ Galleta	44	27,00	20,35 - 34,50	28	63,64	16	36,36	0,002^a^
Bebidas Adobadas	45	27,60	20,90 - 35,15	23	51,11	22	48,89	0,230
Ver televisión	50	31,06	24,01 - 38,82	18	36,00	32	64,00	0,199
Gachas con leche o leche espesadas con harina	36	22,22	16,08 - 29,42	14	38,89	22	61,11	0,553
El niño ha tomado jugo industrializado o polvo de refresco en el último mes.	59	36,42	29,01 - 44,33	21	35,60	38	64,40	0,110
El chico ha tenido soda en el último mes.	47	28,83	22,02 - 36,44	19	40,42	28	59,57	0,608
Niño recibió miel/meldew/azúcar/rapadura antes de los 6 meses de edad	19	11,73	7,21 - 17,71	11	57,89	8	42,11	0,188
El niño recibió alimentos salados antes de la edad adecuada	39	24,22	17,83 - 31,59	15	38,46	24	61,54	0,415

C I (95%): 95% Intervalo de confianza de % * se omitió la variable “no”, se presentaron los datos de la variable “sí” que representa el consumo de alimentos. ^*a*^ *variable asociada al estado nutricional materno.*

*Fuente: Elaboración de los autores*

## Discusión

Un paso importante en la prevención de la obesidad es conocer las causas y determinantes. En el presente estudio, la madre con un estado nutricional inadecuado fue una variable relevante que puede contribuir a la imagen de la obesidad infantil, ya que los niños que tienen madres con un estado nutricional inadecuado son más propensos a verse afectados por trastornos nutricionales con sobrepeso^4^^,^^23^.

Según nuestro estudio, los hábitos nutricionales de los niños se forman en las primeras etapas de sus vidas y dependen de una compleja red de factores fisiológicos y ambientales^24^_Entre los factores ambientales se encuentran las relaciones con padres, familiares, amigos, colegas y otras personas que tienen cierta proximidad con el niño, entre estos grupos destaca la familia, y dentro de esto la madre como la principal influencer en hábitos alimenticios^5^.

En el presente estudio, se pudo observar que el 30,06% de los bebés y el 51,53% de las madres tenían sobrepeso, corroborando los hallazgos en la literatura y evidenciando la transición nutricional que existe en el país, delimitada por la reducción de la prevalencia de la desnutrición y el aumento de la prevalencia de la obesidad. La obesidad es un problema de salud pública en todo el mundo, y que en las últimas décadas han afectado a niños y adolescentes^1^.

El sobrepeso en la infancia conduce a consecuencias negativas a corto y largo plazo y eso puede dificultar su vida social, entre las principales repercusiones se encuentran la aparición de enfermedades crónicas no transmisibles, depresión y ansiedad, muerte temprana, trastornos del sueño^4^ Además, las personas con sobrepeso y obesidad en la infancia y la adolescencia tienden a mantener este estado nutricional a lo largo de su vida^25^.

El factor predisposición para el sobrepeso son los cambios en el estilo de vida, como el estilo de vida sedentario y el alto consumo de alimentos ultraprocesados (AUP) en detrimento del bajo consumo de alimentos *frescos o mínimamente* procesados; y este hecho alteró los hábitos alimenticios de todos, incluido el de los infantes^26^^-^^28^.

La ingesta de AUP no se recomienda para la población en general, especialmente en la primera infancia debido a su composición nutricional, ya que tiene un alto contenido de grasas totales, trans y saturadas, sodio, carbohidratos simples, alto valor energético, bajo contenido de micronutrientes y fibra^29^ Además, cabe destacar que en su proceso de fabricación existen cinco o más ingredientes que suelen ser exclusivos de la industria alimentaria, como sustancias alimentarias y aditivos, lo que los hace incapaces de reproducirse en el entorno nacional^30^ Sobre todo, el consumo de PUA está fuertemente asociado con la presencia de sobrepeso en niños y adolescentesde^31^.

En el presente estudio, la aparición de las AUP en la alimentación infantil fue notable, reforzando que las ultra procesadas están cada vez más presentes en la dieta brasileña y se introducen a tiempo, corroborando el estudio de Lopes et al.^32^ en los que el 74,3% de los 545 menores de 24 meses tenían el consumo de algunos alimentos industrializados. Además, Giesta et al.^9^ en una muestra de 300 niños, 263 ya habían sido presentados a una AUP, y para el 56,5% de estos niños la introducción se produjo antes de seis meses de vida.

En cuanto al perfil materno, la literatura indica una fuerte relación entre la escolarización y los ingresos maternos con la presentación de las AUP en la alimentación infantil, donde las madres con baja escolaridad e ingresos tienen una mayor tendencia a introducir alimentos industrializados a sus hijos, características también coherentes con una muestra de presente estudio^12^^,^^33^. Además, los resultados de Freitas et al.^34^, en un estudio con niños de 1 año, señalan que los bebés cuyas madres tenían una educación más baja tenían una mala o regular calidad de alimentación, en comparación con los niños nacidos de madres con educación secundaria completa.

En el ámbito de las variables estudiadas y de los AUP asociada a un estado nutricional materno inadecuado la ingesta de fideos instantáneos/ merienda/galleta, la literatura indica que existe una mayor similitud entre la alimentación del binomio madre-hijo en comparación con el padre-hijo^35^. Del mismo modo, un estudio con preescolares reveló que los hijos de madres con sobrepeso tenían un menor consumo de frutas, lo que puede favorecer la ingesta de alimentos industrializados y contribuir al sobrepeso de los bebés^36^. Además, la obesidad materna está relacionada con una disminución de la intención de la mujer de amamantar a su hijo, tanto en términos de inicio como de duración de la lactancia materna^13^.

Por último, Canella et al.^37^ al analizar la disponibilidad de AUP en los hogares brasileños utilizando datos recogidos en la Encuesta de Presupuesto Familiar 2008-2009, encontró una asociación positiva entre la prevalencia del sobrepeso y la obesidad y la disponibilidad de AUP en los hogares brasileños.

A pesar de la asociación no estadística, cabe destacar que, en este estudio, los niños cuyas madres tenían insuficiencia nutricional tenían el hábito frecuente de comer viendo televisión, y un mayor consumo de azúcares y refrescos. Bassul et al.^38^ al día a mayores posibilidades de que el bebé consuma productos ricos en azúcares y bebidas endulzadas, y un menor consumo de verduras por parte de los niños. La literatura señala que el hábito de comer asociado a una pantalla o medios de comunicación contribuye a la pérdida de percepción de la saciedad, el estilo de vida sedentario e influye en las opciones alimentarias, promoviendo en el niño una preferencia por las AUP porque están asociadas con personajes y juguetes .

Los procedimientos estadísticos no permitieron extraer una estructura de interacciones para percibir una relación jerárquica entre la asociación del estado nutricional materno con la ingesta de alimentos de niños de entre 6 y 24 meses de edad. Sin embargo, los resultados plantean la cuestión de que el peso de las madres influye en las actitudes de control hacia la dieta de los niños. A su vez, estas actitudes probablemente contribuirán a la promoción de un comportamiento alimenticio característico de los niños y jóvenes, con implicaciones directas en el estado de peso. Esta hipótesis todavía necesita confirmación, y por lo tanto será un camino para explorar.

Los resultados tienen implicaciones para el asesoramiento y la intervención en niños obesos, o en riesgo de obesidad, y las madres. Además, la comprensión de que el comportamiento alimenticio de los niños también depende del comportamiento alimenticio de las madres. Los cambios en los hábitos alimenticios de los niños deben ir precedidos de cambios en el entorno familiar, por lo que es esencial incluir a los padres en el proceso de intervención en la obesidad.

En un momento, el trabajo actual es una herramienta para el conocimiento de la alimentación infantil y factores relacionados, y se convierte en un instrumento para dirigir las actividades de los profesionales de la salud en la atención primaria de salud.

Los resultados del presente estudio deben ser interpretados considerando algunas limitaciones inherentes a la investigación, tales como la información autorreportada y el tipo de estudio serían aspectos. El carácter transversal no permite asociaciones de causa y efecto de los datos recogidos. Otra limitación sería el instrumento de recolección de datos, el formulario de marcadores de consumo de alimentos, ya que busca identificar la calidad de la alimentación del niño, pero no permite cuantificar las porciones de alimentos consumidos.

Se sugiere realizar estudios longitudinales para comprender qué variables y cómo la relación binomio-niño interfiere en la alimentación del lactante y, en consecuencia, en el estado nutricional del lactante.

## Conclusión

Los resultados de este estudio muestran que el estado nutricional de la madre puede ser un factor que contribuya positivamente al sobrepeso infantil. Sin embargo, es importante resaltar que los determinantes del exceso de peso son múltiples y que las relaciones entre ellos no son lineales, pudiendo estar influenciados por factores económicos, sociales y nutricionales, como puede ser el caso de este estudio, dado que la muestra en su mayoría era de escasos recursos y los niños tenían un estado nutricional adecuado. En ese sentido, se sugieren más estudios para comprender y dilucidar los factores que interfieren en la nutrición infantil y materna y, consecuentemente, en el estado nutricional y de salud de esta población. Los programas de intervención para la prevención y tratamiento de este trastorno nutricional son complejos e involucran a varios actores sociales. Por lo tanto, destacamos la importancia de la educación alimentaria y nutricional para promover prácticas de alimentación saludable por parte del binomio madre-hijo.
